# Phytochemicals as Therapeutic Interventions in Peripheral Artery Disease

**DOI:** 10.3390/nu13072143

**Published:** 2021-06-22

**Authors:** Ahmed Ismaeel, K. Leigh Greathouse, Nathan Newton, Dimitrios Miserlis, Evlampia Papoutsi, Robert S. Smith, Jack L. Eidson, David L. Dawson, Craig W. Milner, Robert J. Widmer, William T. Bohannon, Panagiotis Koutakis

**Affiliations:** 1 Department of Biology, Baylor University, Waco, TX 76798, USA; ahmed_ismaeel@baylor.edu (A.I.); Leigh_Greathouse@baylor.edu (K.L.G.); evlampia_papoutsi@baylor.edu (E.P.); 2 Department of Human Sciences and Design, Baylor University, Waco, TX 76798, USA; 3 Department of Chemistry and Biochemistry, Baylor University, Waco, TX 76798, USA; Nathan_Newton1@baylor.edu; 4 Department of Surgery, University of Texas Health Science Center San Antonio, San Antonio, TX 78229, USA; miserlisd@uthscsa.edu; 5 Department of Surgery, Baylor Scott & White Medical Center, Temple, TX 76508, USA; Robert.Smith@BSWHealth.org (R.S.S.); Jack.Eidson@BSWHealth.org (J.L.E.); David.dawson@bswhealth.org (D.L.D.); craig.milner@bswHealth.org (C.W.M.); William.Bohannon@BSWHealth.org (W.T.B.); 6 Heart & Vascular Department, Baylor Scott & White Medical Center, Temple, TX 76508, USA; Robert.Widmer@BSWHealth.org

**Keywords:** polyphenols, beetroot, cocoa, flavonols, claudication, hindlimb ischemia

## Abstract

Peripheral artery disease (PAD) affects over 200 million people worldwide, resulting in significant morbidity and mortality, yet treatment options remain limited. Among the manifestations of PAD is a severe functional disability and decline, which is thought to be the result of different pathophysiological mechanisms including oxidative stress, skeletal muscle pathology, and reduced nitric oxide bioavailability. Thus, compounds that target these mechanisms may have a therapeutic effect on walking performance in PAD patients. Phytochemicals produced by plants have been widely studied for their potential health effects and role in various diseases including cardiovascular disease and cancer. In this review, we focus on PAD and discuss the evidence related to the clinical utility of different phytochemicals. We discuss phytochemical research in preclinical models of PAD, and we highlight the results of the available clinical trials that have assessed the effects of these compounds on PAD patient functional outcomes.

## 1. Introduction

Although modern nutritional science is thought to date back to only the 20th century (with the discovery of vitamins), phytochemicals, or plant-derived chemicals, have been used traditionally in medicine for thousands of years across the world [1]. In fact, the Greek physician Dioscorides wrote a pharmacopoeia of medicinal plants, *De materia medica*, as early as the year 50 AD [2]. Following the era of nutrient discovery with a focus on deficiency diseases, the latter half of the 20th century saw nutritional research more focused on identifying the relationship between food, nutrition, and chronic diseases [1,3]. Large epidemiologic studies have consistently demonstrated inverse relationships between fruit and vegetable consumption and the risk of a wide range of chronic diseases as well as all-cause mortality [4,5,6]. This relationship has been largely studied in the context of coronary heart disease and stroke, and higher fruit and vegetable intake are consistently associated with reduced risk of these cardiovascular/cerebrovascular diseases [7]. The most common underlying cause of cardiovascular and cerebrovascular disease is atherosclerosis, a disease involving multiple factors including inflammation, oxidative stress, endothelial dysfunction, and dyslipidemia [8]. Interestingly, subclinical measures of atherosclerosis are also inversely associated with fruit and vegetable intake [9], and in animal models, fruit and vegetable supplementation can reduce subclinical markers of atherosclerosis [10]. Another atherosclerotic disease, peripheral artery disease (PAD), affects arteries other than those supplying the brain or heart, most commonly affecting the femoropopliteal or infrapopliteal arteries of the legs [11,12]. Although there has been less research on dietary composition and PAD, the available data suggest a similar association of reduced PAD risk with higher fruit and vegetable intake. One cross-sectional study including over 3.6 million participants showed that consumption of 3 or more servings of fruits and vegetables per day was associated with 18% lower odds of PAD than consuming ≥3 servings of fruits and vegetables less than once a month [13]. Likewise, in a case-cohort study of 944 patients with type 2 diabetes, a high-score Mediterranean dietary pattern (diet rich in plant-based foods and polyphenol-rich olive oil) was independently associated with a 56% reduced risk of PAD [14]. These data suggest that fruits and vegetable consumption may be a lifestyle and dietary risk factor for PAD.

The mechanisms behind the health benefits associated with fruits and vegetables are thought to be largely attributable to phytochemical components of plants [15]. There have been thousands of identified phytochemicals which are divided into classes based on their chemical structures that largely dictate their function [16]. These categories broadly include flavonoids, stilbenes, organosulfur compounds, nitrogen-containing compounds, carotenoids, and diarylheptanoids [16]. The main mechanism by which phytochemicals are thought to exert their health-promoting effects is via antioxidant effects. Although some phytochemicals possess free radical scavenging ability in vitro [17], direct reaction with reactive oxygen species (ROS) in vivo is less likely due to the lower dose of the phytochemicals in vivo. For example, phytochemical concentrations in plasma are around ~1 µM, and the levels in tissue are not believed to exceed the nanomolar range, while antioxidant enzymes such as superoxide dismutase can be present at concentrations as high as ~20 µM [18]. Instead, phytochemicals can act to induce de novo expression of endogenous antioxidant defense genes, such as glutathione peroxidase, superoxide dismutase, NAD(P)H quinone oxidoreductase, and heme oxygenase-1 [16]. These genes contain a common antioxidant-responsive element in their 5′-flanking regions, which can be bound and regulated by transcription factors such as nuclear factor erythroid 2-related factor (Nrf2) or the aryl hydrocarbon receptor [19,20]. Various phytochemicals have been shown to enhance the expression and activation of these transcription factors, thus inducing the activation of target antioxidant enzymes [16]. In addition to these mechanisms, organosulfur, and nitrogen-containing phytochemicals are thought to exert unique protective effects by increasing nitric oxide and hydrogen sulfide bioavailability [21]. Both nitric oxide and hydrogen sulfide are important gaseous transmitters that work together to induce smooth muscle relaxation, vasodilation, increased blood flow, and angiogenesis [22], and hydrogen sulfide is also a known activator of Nrf2 [23]. Notably, PAD patients have been shown to have reduced levels of hydrogen sulfide, reduced Nrf2 activation, and reduced NO bioavailability compared to non-PAD controls [24,25]. Modulation of these pathophysiological processes by phytochemicals may be viable therapeutic options for PAD patients.

Phytochemicals have been studied in a wide range of conditions ranging from cancer [26] to musculoskeletal disorders [27]. Likewise, previous reviews have focused on the potential cardiovascular and cerebrovascular beneficial effects of phytochemicals [28,29]. However, no comprehensive review article has reported on the preclinical and clinical vascular beneficial effects of these compounds in the context of limb ischemia, a major complication of PAD. Thus, the purpose of this narrative review is to summarize the research assessing the effectiveness of different phytochemicals as therapeutic interventions in the context of PAD. We include studies on phytochemicals that have been studied in preclinical models of the disease as well as in clinical trials including PAD patients.

## 2. Peripheral Artery Disease

PAD is an atherosclerotic condition of the extremities, mostly affecting arteries of the legs [12]. According to estimates, over 200 million people globally are affected with PAD [30]. PAD is especially common in older individuals, affecting up to 27% of 45- to 74-year-olds and 1 in 3 individuals in the 91–100-year-old age group [31,32]. Based on the Fontaine classification system, asymptomatic PAD patients are classified in Stage I, and patients presenting with the most common symptom of PAD, intermittent claudication, (walking-induced leg pain) are classified in Stage II. In the later stages of PAD, patients with foot pain at rest are classified in Stage III, and Stage IV patients with ulcers and gangrene are considered to have critical limb ischemia [33].

PAD is associated with an elevated risk of all-cause mortality, cardiovascular disease mortality, myocardial infarction, and stroke [34,35,36]. In addition, PAD patients experience exercise limitations and dramatic reductions in walking performance of up to 50% of non-PAD controls [37,38,39]. The pathophysiology of the functional impairment found in PAD is believed to encompass different mechanisms, including oxidative stress [40], skeletal muscle myopathy [41], and endothelial dysfunction [42]. Increased oxidative stress markers have been documented in PAD, and oxidative stress markers are correlated with reduced walking distances of patients [43,44]. Likewise, nitric oxide levels are reduced in PAD [24], and flow-mediated dilation (FMD), a measure of endothelial function, is associated with patients’ walking ability [45]. Notably, inflammation is also thought to play a role in the pathophysiology of PAD [46]. Several inflammatory markers have been shown to have a strong predictive value for the development and presence of PAD [46]. Large cohort studies have shown that markers of inflammation such as C-reactive protein (CRP) and cellular adhesion molecules are independently associated with PAD risk [47,48]. In addition to their role as prognostic indicators; however, inflammatory molecules are also implicated in disease mechanisms. In fact, atherosclerosis has been described as an “inflammatory disease,” with evidence suggesting that components of inflammation in the arteries can lead to progressive arterial damage [49]. Inflammatory cytokines such as tumor necrosis factor alpha (TNF-α) and interleukins (ILs) can lead to arterial stiffening via increased leukocyte infiltration, enhanced elastin degradation, and increased vascular smooth muscle cell de-differentiation and collagen synthesis [50]. Immune cells recruited by inflammatory cytokines can also alter oxidative balance, as these cells are known to be major sources of ROS [51]. Furthermore, inflammatory molecules such as CRP can directly inhibit endothelial NO synthase activity [52]. CRP can also promote production of superoxide, which can result in NO synthase uncoupling by oxidation of the critical NOS co-factor, tetrahydrobiopterin [53]. Therefore, oxidative stress, inflammation, endothelial dysfunction, and the interactions between these processes are important considerations in PAD pathogenesis.

Without therapeutic interventions, PAD also leads to significantly accelerated functional decline over time [54]. Thus, although the primary treatment goals for patients is to improve cardiovascular risk and limb salvage, improving exercise performance, daily functional and physical activity, and quality of life are also important treatment considerations.

Recent research on PAD interventions have focused on targeting oxidative stress, skeletal muscle pathology, and nitric oxide bioavailability to improve functional outcomes in patients. For practical significance, the minimal clinically important differences in walking performance following an intervention for PAD patients have been defined. For the 6-min walk test, a small meaningful change is considered an improvement in walking distance of 8–20 m, and a large meaningful change has been defined as a change of 50 m [55,56]. During a treadmill test, an increase in peak walking time of at least 38 s and an increase in claudication onset time of at least 35 s have been suggested as small minimal clinically important differences [57]. Currently, treatment options for PAD management are limited with only two modestly effective medications approved for claudication [58,59]. On the other hand, supervised exercise training has been shown to lead to greater improvements in walking performance in PAD [60]. Despite the success of individual interventions, the long-term efficacy of all treatment options for PAD remains questionable. For example, an interesting recent network meta-analysis of 46 randomized controlled trials compared the short-, moderate-, and long-term outcomes of a variety of treatments for PAD including supervised exercise therapy, endovascular revascularizations, and cilostazol [61]. It was found that although some treatments effectively improved walking distance at short- or moderate-term follow-up, none of the treatments improved walking performance of PAD patients in the long-term (≥2 years) [61]. This highlights the dire need for more durable PAD treatments.

In addition, exercise bouts have been shown to increase ROS biomarkers in PAD patients, and some studies have shown that exercise can lead to pathologic changes in skeletal muscle and skeletal muscle injury [62,63,64]. This observation has led some researchers to suggest the addition of antioxidant supplementation along with exercise interventions to alleviate exercise-induced oxidative stress [65,66]. Although some smaller studies have suggested that nonspecific antioxidants such as vitamin C and vitamin E may improve exercise tolerance in PAD patients [65,67,68], large-scale trials such as the “POPADAD” trial and the “HOPE” trial found no evidence that these antioxidants can improve outcomes such as revascularizations, amputations, and cardiovascular events [67,68]. Some researchers argue that this may be due to the fact that the concentration of vitamins C and E that would be necessary to induce antioxidant effects in the vascular wall may be unachievable, neither vitamin reacts appreciably with hydrogen peroxide, and the vitamins themselves may also be oxidized and inactivated by free radicals [69,70]. Instead, a better strategy may be therapies with antioxidant concepts such as phytochemicals that act to increase antioxidant capacity by activation of endogenous antioxidant defense systems. Thus, in recent years, a number of preclinical studies and a few clinical trials have sought to test the efficacy of individual plant components for the mitigation of the oxidative stress and oxidative-stress-induced damage and pathology associated with limb ischemia. These studies are discussed in the next sections.

## 3. Preclinical Research

### 3.1. Hindlimb Ischemia

Preclinical murine models of hindlimb ischemia (HLI) have long been used to simulate PAD pathophysiology [71]. The most common method for induction of HLI is by ligation or excision of the femoral artery [72]. However, this approach is considered an acute limb ischemia model, and blood flow recovers rapidly due to the quick formation of collateral circulation [73]. Thus, other approaches have been developed, such as gradual femoral artery occlusion with ameroid constrictors (subacute) [74] and a two-stage limb ischemia model (chronic), which are considered more relevant to the human disease [75,76]. HLI models typically result in reduced limb perfusion, functional deficits, reduced walking performance, systemic inflammation, and skeletal muscle damage [73]. However, it is important to note that other manifestations that play an important role in PAD pathogenesis may not be well-modeled in animal HLI studies. For example, PAD can be accompanied by ulcers; yet, an animal model of non-healing ulcers in limb ischemia has not yet been established [77]. In fact, very few studies have demonstrated wound generation in the clinically relevant peripheral site of the limb or in older animals, and other issues related to study design and bias reduction also exist [77]. It is also important to note that the HLI induction technique as well as other factors, including species (rat vs. mouse), strain, age, and the presence of other comorbid conditions, can affect the resulting ischemic damage [74]. Regardless of the procedure, HLI models are considered useful for testing new therapies for PAD treatment [73], and have thus been used to study a wide range of potentially beneficial phytochemicals.

### 3.2. Flavonoids

Flavonoids are a category of polyphenolic metabolites found in plants, characterized by a general structure of a 15-carbon skeleton, consisting of 2 phenyl rings and a hetrocyclic, oxygen-containing ring [78]. Flavonoids are further categorized into six subgroups, the anthocyanidins, anthoxanthins, flavanones, flavanonols, flavans, and isoflavonoids [79]. The results of a meta-analysis of flavonoid effects on aortic atherosclerosis in mice suggest that flavonoids can significantly reduce aortic atherosclerosis [80]. Interestingly, atherosclerosis area-reducing effects were isolated to flavonols, but not flavan-3-ols, suggesting that flavonols may hold the most potential therapeutic value in atherosclerosis [80].

**Figure 1:**
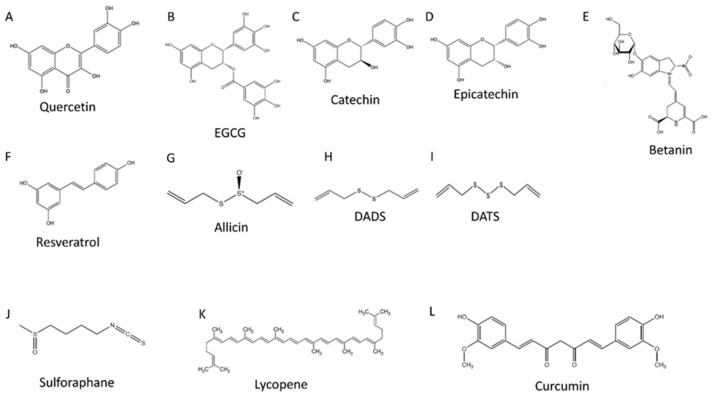
Polyphenol chemical structures. (**A**) is the chemical structure of quercetin, (**B**) is the chemical structure of epigallocatechin gallate (EGCG), (**C**) is the chemical structure of catechin, (**D**) is the chemical structure of epicatechin, (**E**) is the chemical structure of betanin, (**F**) is the chemical structure of resveratrol, (**G**) is the chemical structure of allicin, (**H**) is the chemical structure of diallyl disulfide (DADS), (**I**) is the chemical structure of diallyl trisulfide (DATS), (**J**) is the chemical structure of sulforaphane, (**K**) is the chemical structure of lycopene, and (**L**) is the chemical structure of curcumin.

Quercetin (Figure 1A), an anthoxanthin, is a flavonoid found in many fruits and vegetables, with the most appreciable amounts found in red onions and kale [81]. Quercetin has been well-studied in basic research due to its antioxidant potential [82]. In rats undergoing acute HLI, 200 mg/kg/day of quercetin, administered for one week prior to ischemia induction, significantly increased antioxidant enzyme activity and reduced lipid peroxidation markers [83]. However, in contrast to this study, a more recent study assessed the effect of quercetin (~185 mg/kg/day for 30 days following HLI) in the sustained, two-stage model of HLI [84]. These mice also had dyslipidemia (apolipoprotein E-deficient), which further simulates human PAD [76]. Utilizing this model, quercetin had no effect on limb perfusion. Despite this, plasma nitrate and nitrite concentrations were significantly increased in mice that consumed quercetin at the end of the study, although oxidative stress markers were not measured [84]. Furthermore, this study also measured the clinically relevant variables of exercise performance and physical activity on a treadmill as well as an open field test, and quercetin was shown to have no effect on distance travelled or velocity during either test [84]. Therefore, although it may have some effect on reducing oxidative stress markers, due to the lack of improvement in functional performance during sustained HLI, quercetin is unlikely to be effective for PAD treatment. Notably, this ineffectiveness may be due to its low bioavailability. In another study, quercetin glucosides (enzymatically trans-glycosylated isoquercitin), which have a higher solubility in water and better bioavailablity compared with quercetin, were administered to mice (100 mg/kg/day) for 2 weeks prior to acute HLI induction and 2 weeks following surgery [85]. Blood flow recovery and capillary density were augmented in ischemic limbs of mice that received quercetin glucosides, along with plasma glutathione levels [85]. Thus, conversion of quercetin into glucosides may be a promising strategy to enhance its bioavailability and effectiveness in future interventions.

The flavan subgroup of flavonoids include catechin, epicatechin, and epigallocatechin 3-gallate (EGCG) [79]. EGCG (Figure 1B) is highly abundant in green tea and is believed to be responsible for much of the effects mediated by tea [86]. Although EGCG administration (50 mg/kg, administered 30 min prior to reperfusion) was shown to reduce oxidative status and increase total antioxidant status following acute HLI-reperfusion in rats (2 h), EGCG had no protective effect on oxidative or antioxidant status following long-term reperfusion (24 h), which better resembles true ischemia-reperfusion conditions [87]. Furthermore, serum inflammatory cytokine levels (IL-1, IL-6, IL-8, and TNF-α) were unaffected by EGCG administration in either short- or long-term reperfusion [87]. EGCG, due to its pyrocatechol structure, has been shown to coordinate with metal ions, and these metal-EGCG networks have also been applied to the study of different conditions [88,89]. In mouse HLI models established by femoral artery ligation and excision, copper-EGCG capsules or zinc-EGCG capsules injected into ischemic muscles reduced ischemic damage and improved blood flow recovery, vessel volume, and angiogenic markers [90,91]. However, there were no significant differences with respect to the levels of the inflammatory cytokines TNF-α and IL-6. Therefore, although these data suggest that metal-EGCG coordinated capsules may hold promise as potential medical therapies, their safety and efficacy must first be established in humans. Additionally, it is unclear how much of the benefit was due to the metal compared to EGCG. Thus, there is not enough evidence to suggest that EGCG alone may be an effective treatment for PAD.

Catechin (Figure 1C) and epicatechin (Figure 1D) are found in many different foods including prune juice, peaches, tea, and açaí. Notably, cocoa is believed to have the highest content of catechins among all foods (>100 mg/100 g) [78]. Catechin and epicatechin have been heavily studied in different preclinical models. In one in vitro study, supernatant of activated platelets from PAD patients were used to increase soluble cell adhesion molecules (markers of endothelial activation and inflammation) and reduce NO bioavailability in human umbilical vein endothelial cells [92]. Pretreatment with 0.1–10 µM of catechin and epicatechin was shown to reduce the increase in adhesion molecules induced by PAD platelets and increase NO levels [92]. Furthermore, in a mouse HLI model induced by two ligations proximal and distal to the femoral artery, epicatechin feeding, starting at 5 days prior to ischemia induction, improved perfusion recovery and capillary density in a dose-dependent manner, with maximal effects seen at 2 mg/kg per day [93]. These promising preclinical results have led to a number of clinical trials in PAD patients using catechin- and epicatechin-rich dark chocolate/cocoa, which will be discussed in later sections.

### 3.3. Nitrogen-Containing Compounds

Certain phytochemicals that contain nitrogen atoms provide a dietary source of inorganic nirate, which been suggested to have potential beneficial effects related to modulation of NO levels [94]. Nitrate-containing vegetables, such as spinach, arugula, celery, and beetroot, have been thought to be key mutual components of diets associated with low incidences of cardiovascular disease (i.e., Mediterranean and Japanese traditional diets [94]). Notably, nitrate therapy has been showed to induce pro-angiogenic effects in several models of HLI, including murine [95,96] and swine [97,98]. Of all the studied dietary nitrate donors, beetroot (Beta vulgaris) is one of the most heavily researched [94]. Aside from the nitrate-mediated effects of beetroot, associated health benefits may also be a consequence of the properties of the betanin pigment found in beetroot (Figure 1E). Betanin is one of the betalain pigments, which are a group of red/yellow pigments found in plants, chemically characterized as tyrosine-derived aromatic indoles [99]. Compounds with an indole moiety are known to possess antioxidant activity and likely activate the aryl hydrocarbon receptor [100]. A number of studies have also suggested that betalains may possess strong antioxidant and anti-inflammatory properties [101,102,103]. During the last 10 years, beetroot has been extensively studied in preclinical and clinical research for its potential cardiovascular benefits [104]. However, beetroot has yet to be studied in any animal models of limb ischemia. In a cardiovascular model, mice were exposed to coronary artery occlusion for 30 min followed by 24 h of reperfusion as a model of cardiac ischemia-reperfusion [105]. Interestingly, mice given beetroot juice powder at a dosage containing 0.7 mM nitrate showed reduced infarct size and improved ventricular function [105]. This suggests that beetroot may play a protective role in the context of ischemia-reperfusion injury, including limb ischemia, but studies in a HLI model are required before drawing definite conclusions.

### 3.4. Stilbenes

Stilbenoids are hydroxylated derivatives of 1,2-diphenylethene (stilbene), characterized by a C6-C2-C6 structure [106]. One of the most widely studied stilbenoids is 3,5,4′-trihydroxy-trans-stilbene, or resveratrol (Figure 1F), which is known for its content in red wine as well as red or purple grapes. Early in vitro data and in vivo work suggested that resveratrol may hold therapeutic potential for lifespan extension as well as the prevention or slowed progression of different diseases, including cancer, cardiovascular disease, and diabetes [107,108,109]. While the exact mechanisms are unclear, resveratrol has been shown to exert potent antioxidant effects by modulating antioxidant enzymes and intrinsic antioxidant capacity [110]. Furthermore, resveratrol is a known activator of sirtuin 1, a histone deacetylase enzyme that regulates expression of genes involved in metabolic regulation, survival, autophagy, and stress response [110].

In acute rat models of HLI, administration of 1–20 mg/kg/day resveratrol was shown to reduce oxidative stress markers, improve hindlimb skeletal muscle histology (reduced myofiber atrophy, segmental necrosis, and edema), and protect femoral artery tissue from vascular wall thickening, apoptosis, inflammatory cell infiltration, and edema [111,112,113]. In rabbits with HLI induced by femoral artery excision, resveratrol (2.5 mg/day) has also been demonstrated to have pro-angiogenic effects by improving endothelial NO synthase expression, limb blood flow, and capillary density [114]. Similar effects were shown after injection of bone marrow derived nuclear cells incubated with 100 µM resveratrol into hindlimb muscles of mice with HLI induced by femoral artery excision [115]. Thus, in preclinical models, resveratrol has shown efficacy in improving ischemic pathology.

### 3.5. Organosulfur Compounds

Organosulfides refer to sulfur-containing organic compounds, which are classified according to the sulfur-containing functional group. One of these compounds is allicin (Figure 1G), the active flavor compound in chopped or crushed garlic [116]. After formation, allicin is quickly converted into compounds including diallyl disulfide (DADS) (Figure 1H) and diallyl trisulfide (DATS) (Figure 1I) [117]. Importantly, both DADS and DATS have been shown to play a role in cellular detoxification and protection against oxidative stress, and DATS is further known to be a source of hydrogen sulfide [118]. Thus, DATS has been widely studied in the context of ischemia-reperfusion injury [119]. In a mouse model of HLI induced by femoral artery ligation, injection of 500 µg/kg/day i.p. DATS for 10 days improved blood flow recovery, capillary density, and NO bioavailability [120]. These results were also duplicated in mice with concomitant HLI and streptozotocin-induced diabetes [121].

Another promising organosulfur compound is sulforaphane (Figure 1J), a compound found in cruciferous vegetables such as broccoli that is known to be a potent Nrf2 activator [122]. Importantly, sulforaphane has been shown to protect against ischemia-reperfusion injury in preclinical models of cerebral [123], retinal [124], intestinal [125], myocardial [126], and renal [127] ischemia. Sulforaphane has also recently been studied for its potential impact on cardiac and skeletal muscle dysfunction associated with aging [128]. In older mice (21–22 months old), feeding 442.5 mg/kg/day of sulforaphane for 12 weeks restored the age-associated loss in mitochondrial function, cardiac function, skeletal muscle satellite cell activation and differentiation, and skeletal myofiber cross-sectional area [128]. These changes were also accompanied by improved exercise performance, assessed as improved running time and work until exhaustion during a treadmill test and improved isometric grip strength [128]. Sulforaphane may also play a role in the attenuation of atherosclerosis. In a streptozotocin-induced diabetic mouse model, feeding 0.5 mg/kg/day of sulforaphane for 12 weeks prevented the diabetes-associated aortic fibrosis [129]. Likewise, in rabbits fed a high cholesterol diet, supplementation of the diet with 0.25 mg/kg/day of sulforaphane reduced fibrosis and edema in aortic tissue, normalized NO levels, and improved endothelial function [129]. Although sulforaphane has not been specifically studied in the context of HLI, studies from other in vivo models of similar pathophysiology suggest that sulforaphane may be a promising therapeutic in the context of limb ischemia.

### 3.6. Carotenoids

Carotenoids are yellow, orange, or red pigments produced mainly by plants and algae. Their general structure is characterized by a polyene hydrocarbon chain, consisting of 4 terpene units each containing 10 carbon atoms [130]. Carotenoids are further divided into oxygenated (xanthophylls) and oxygen-free subclasses (carotenes). Carotenes include α-carotene, β-carotene, and lycopene. Both α- and β-carotene are considered precursors to Vitamin A (provitamin A) [130]. Interestingly, despite the antioxidant properties of carotenes, two large cross-sectional studies (The Rotterdam study, N = 4367 and The Edinburgh Artery Study, N = 1592) both showed no association between dietary intake of β-carotene and risk of PAD or lower extremity blood flow [131,132]. Although no studies have assessed the effectiveness of α- or β-carotene supplementation in preclinical or clinical limb ischemia, lycopene (Figure 1K) has been studied in this context. In rats with HLI, induced by clamping of the abdominal aortae, feeding 10 mg/kg/day for 15 days prior to ischemic induction protected hindlimb skeletal muscles from atrophy and necrosis [133]. Lycopene has also been shown to protect against injury due to other forms of ischemia, including myocardial [134] and cerebral [135] ischemia. However, it is important to note that issues related to lycopene tissue bioavailability may limit its clinical utility [136,137]. The low bioactivity may explain the lack of effectiveness of lycopene consumption on cardiovascular markers in humans [138]. Future studies should take into account factors that may affect the absorption, bioavailability, and bioconversion of specific carotenoids.

### 3.7. Diarylheptanoids

Diarylheptanoids are a group of secondary metabolites of plants, consisting of two aromatic rings connected by a seven carbon chain [139]. Curcuminoids are linear diarylheptanoids, and the most widely studied is curcumin (Figure 1L), isolated from the turmeric flowering plant of the ginger family. Curcumin has been studied in numerous laboratory and clinical studies of several different diseases, including HLI models. In mice with HLI induced by a double ligation of the femoral, great saphenous, and iliac circumflex arteries with a suture, injection of 100 mg/kg i.p. of curcumin 1 h prior to ligation reduced skeletal muscle fibrosis and enhanced muscle fiber density [140]. These effects also translated to enhanced running capacity during a treadmill test in curcumin-treated mice [140]. Curcumin has also been shown to elicit pro-angiogenic effects in HLI. In mice with HLI induced by femoral artery ligation and excision, feeding 1000 mg/kg/day of curcumin for 2 weeks improved perfusion recovery and increased capillary density [141]. This was also demonstrated in mice exposed to the same HLI conditions and concomitant streptozotocin-induced diabetes [142]. However, despite these promising preclinical findings, curcumin’s low bioavailability and limited tissue distribution limit its bioactivity and clinical effectiveness [143]. This has led researchers to call curcumin “deceptive” and “a cautionary tale” [144]. In fact, despite >120 clinical trials of curcumin in several different disease conditions, no double-blinded, placebo controlled clinical trial has been successful [143]. Thus, curcumin is likely not a viable approach for PAD treatment. Table 1 summarizes the results of the preclinical studies discussed.

### 3.8. Phytochemical Bioavailability

One of the issues related to the clinical utility of phytochemicals is that of bioavailability. While certain compounds can demonstrate promising results in vitro or in animal models, these effects do not always translate to human studies [145]. Cell culture studies only assess biological effects, without taking into account the important factors of digestion, absorption, metabolism, and tissue distribution [146]. Moreover, animal studies often use supra-physiological dosages of phytochemicals that are beyond what can be achieved with normal human dietary intakes [147]. In addition, differences in animal and human metabolism may also lead to differential results. For example, there are known variations in the metabolites that result from the consumption of phenols between rodents and humans [148]. Importantly, many factors can influence bioavailability in vivo. One of the most important determinants for a compound’s bioavailability is related to its chemical structure. For example, according to Lipinski et al.’s ‘rule of 5′, compounds with more than five hydrogen-bond donors, 10 hydrogen-bond acceptors, a molecular weight over 500 daltons, and a common logarithm of the partition coefficient (log*P*, a measure of lipophilicity) greater than 5, can be considered to have poor absorption [149]. Certain phytochemicals, such as curcumin, fall into this category. Still, other compounds that would appear to have better bioavailability can demonstrate high excretion rates, low stability in the gastric environment, increased oxidation, rapid first-pass metabolism or high hepatic uptake, extensive metabolism by the gut microbiome, or poor absorption across the intestinal wall [150,151]. Finally, in addition to these factors, the amount of the compound that accumulates at the target site (for example, in the arterial wall) is also another important factor for consideration [152]. Taken together, these issues urge caution when interpreting preclinical studies, and highlight the need for well-controlled human intervention trials to corroborate findings from in vitro and animal models.

## 4. Clinical Research

### 4.1. Flavonoids

Flavonoid intake has been inversely associated with coronary heart disease in several studies [153,154]. Total flavonoid intake is also associated with a lower risk of PAD hospitalizations, atherosclerosis, and aneurysm, and a lower incidence of revascularizations, endovascular surgery, and amputations [155]. Due to the high flavonoid content of cocoa and dark chocolate, the effects of dietary consumption of cocoa and cocoa products on human health has been widely researched. Three previous studies have tested cocoa in the setting of PAD. In one of these studies, the primary outcome measure assessed was flow-mediated dilation (FMD) [156]. In a randomized, controlled, cross-over design, 21 Stage II PAD patients were randomized to either 50 g dark chocolate (70% cocoa content, 13.5 mg catechin, and 45 mg epicatechin) or cocoa-free chocolate (control). FMD was measured at baseline and 2 h after ingestion. There were no significant changes in FMD in either group after chocolate ingestion, and there was no difference in the change in FMD between study arms [156]. The other two studies assessed walking performance as the primary outcome measures following cocoa consumption. Specifically, the first study sought to assess the acute effect of cocoa on walking ability [157]. Twenty Stage II PAD patients received either 40 g dark chocolate (>85% cocoa) or 40 g milk chocolate (control, <35% cocoa) in a randomized, controlled, cross-over design. The maximum walking distance and maximum walking time until maximum claudication pain were assessed during a treadmill test at baseline and 2 h after chocolate ingestion. Maximum walking distance improved significantly (*p* < 0.001) by 11.5 m (+11%) and maximum walking time increased (*p* < 0.001) by 20 s (+15%) following dark chocolate ingestion, but not after milk chocolate ingestion [157]. Additionally, in contrast to the previous study demonstrating no acute change in FMD, in this study FMD was shown to increase significantly after dark chocolate compared to control.

Recently, the results of the COCOA-PAD phase 2 randomized clinical trial were also published [158]. In this study, 44 PAD patients were randomized to either a cocoa beverage containing 15 g of cocoa (75 mg epicatechin) or a placebo beverage daily for 6 months. The primary outcome measures assessed were the change in the maximum walking distance during a 6-min walk test, measured 2.5 h and 24 h after beverage consumption at the 6-month follow-up. At the end of the study, compared with placebo, the 6-min walk distance significantly improved in the cocoa-treated group by 42.6 m at 2.5 h. The 6-min walk distance also increased by 18 m at 24 h [158]. However, there was no significant effect of cocoa on FMD [158]. Notably, increases of 18 m and 42.6 m are both considered meaningful changes in walking performance. However, the data regarding the effect of cocoa flavonoids on FMD are less consistent. For example, although Loffredo et al. demonstrated an increase in FMD following acute dark chocolate consumption [157], Hammer et al. showed no change [156]. One possible explanation may be the higher cocoa content in Loffredo et al.’s study (>85%) compared to Hammer et al.’s study (70%). However, another potential explanation is the lower baseline FMD in Loffredo et al.’s study (2.3%) compared to Hammer et al.’s study (5.1%). Notably, in McDermott et al.’s study, 6 months of cocoa also did not affect FMD, and the mean baseline FMD was 6.47% [158]. Therefore, dark chocolate/cocoa may improve PAD patients’ FMD only when baseline FMD is low (<3%). Overall, the results of these studies suggest a possible therapeutic effect of cocoa flavonoids on walking performance in PAD patients. Future studies in larger samples should be conducted to definitively determine the effect of cocoa flavonoids on endothelial function and walking performance in PAD patients.

### 4.2. Nitrogen-Containing Compounds

In recent years, there has been a specific interest in studying the potential health effects of beetroot. In fact, beetroot has been recommended as an adjunct treatment that may improve clinical outcomes in cardiovascular disease management [159]. Two previous studies have investigated the use of beetroot in the setting of PAD. The first study was a randomized, cross-over study in which 8 Stage II PAD patients consumed 500 mL of beetroot juice or placebo [160]. The primary outcome measures assessed were claudication onset time and peak walking time during a Gardner treadmill test performed 3 h after beverage consumption. Participants walked for 32 s (18%) longer before the onset of claudication following beetroot juice consumption, which was a significant difference compared to placebo. Peak walking time also increased by 65 s (17%) after beetroot juice compared to placebo. [160]. The NO-PAD trial was an extension of this previous preliminary study [161]. In a randomized, per-protocol study design, 24 Stage II PAD patients were assigned to a 36-session (12 week) exercise rehabilitation program along with an oral inorganic nitrate beverage (4.2 mmol beetroot juice), or the exercise program and a placebo. The exercise program consisted of supervised treadmill training for 30 min three times per week. The beetroot juice or placebo were consumed 3 h before training [161]. The primary outcome measures were claudication onset time during a maximal treadmill test and 6-min walk distance. Although the claudication onset time improved for both groups after 12 weeks, the increase was statistically significant in only the exercise + beetroot juice group, resulting in a medium-large effect size of 0.62 [162]. Similarly, the 6-min walk distance improved in both groups, but the increase was only statistically significant in the exercise + beetroot juice group, resulting in an effect size of 0.43 [162]. Notably, a combination of beetroot and exercise improved claudication onset time by 121.1 s (more than one full stage of the graded treadmill test), which was an increase of 200% compared to exercise alone [162]. The increase of over 2 min can be considered a moderate minimal clinically important difference. Thus, based on these pilot studies, beetroot may be a viable treatment option for improving functional performance in PAD patients, especially in combination with exercise. Future study should be conducted in a larger sample to confirm whether beetroot therapy may provide clinically meaningful changes in functional performance.

### 4.3. Stilbenes

Despite many promising preclinical studies, there is little evidence of health benefits of resveratrol in humans due to the poor bioavailability of the compound [163]. Still, some clinical studies have shown some cardioprotective effects of resveratrol, such as improved endothelial function, ventricular and diastolic function, and improved blood lipids [164]. Resveratrol was also studied for its potential impact on walking performance in PAD patients during the RESTORE randomized clinical trial [165]. In this study, 66 patients were randomized to receive 125 mg/day resveratrol, 500 mg/day resveratrol, or placebo for 6 months. The maximum walking time during a 6-min walking test increased only in the group receiving 125 mg/day by 4.6 ± 8.1 m, which was a statistically significant difference based on the defined level of statistical significance (although not considered to be a clinically meaningful improvement). Further, there was no change in the maximum walking time during a treadmill test in either group [165]. Thus, there seems to be no consistent evidence that resveratrol improves walking performance in PAD patients to a clinically relevant extent.

### 4.4. Organosulfur Compounds

Organosulfur compounds, including garlic-derived allicin/DATS, as well as isothiocyanates such as sulforaphane, have been studied in a variety of conditions ranging from autism to metabolic disease and diabetes. However, these compounds have not been well studied in PAD patients. Only one early randomized clinical trial in 80 Stage II PAD patients investigated the effect of garlic powder (800 mg diallyl dose) in combination with a physical therapy training program for 12 weeks on walking performance, compared with the training program and a placebo [166]. Although pain-free walking distance during a treadmill test increased in both groups (physical therapy + garlic: 46 m [28.5%] increase, physical therapy + placebo: 31 m [18.1%] increase), the improvement was significantly greater in the group receiving the garlic powder along with the physical therapy training [166]. The increase of walking distance of 46 m following physical therapy + garlic is considered a clinically meaningful change. Additionally, even the difference in the improvement in walking performance between the patients randomized to physical therapy + garlic compared to physical therapy + placebo of 15 m is a meaningful difference [166]. Table 2 summarizes the results of the discussed clinical trials.

Although sulforaphane has not been specifically tested in PAD patients, clinical trials from other conditions have shown improvements in atherosclerosis-related biomarkers following supplementation. Specifically, two independent randomized controlled trials (*N* = 130) reported that consumption of high sulforaphane-content broccoli (400 g broccoli, containing 24.83 µmol/g glucoraphanin, the glucosinolate of sulforaphane) for 12 weeks reduced plasma LDL-C [167]. Likewise, in 40 overweight participants, consumption of 30 g/day of sulforaphane-containing broccoli sprouts (51.08 mg glucoraphanin) reduced the inflammatory markers interleukin-6 and C-reactive protein [168]. Additionally, one of the most promising applications of sulforaphane to date has been in the treatment of type 2 diabetes. An earlier 4-week randomized controlled trial (*N* = 63) administering 112.5 or 225 μmol/d sulforaphane to patients with diabetes showed significant reductions in oxidative stress markers and oxidized LDL [169]. This was followed by other randomized controlled trials (*N* = 72) that demonstrated that the same protocol of sulforaphane administration reduced serum triglycerides and the atherogenic index of plasma [170], and decreased serum insulin concentration and improved insulin resistance [171]. Most recently, a 12-week study administering 150 μmol sulforaphane/day in 103 obese patients with type 2 diabetes showed that sulforaphane improved fasting glucose and glycated hemoglobin [172]. One of the aspects related to potential organosulfur compound benefits may be the comparatively high bioavailability. For example, in contrast to a number of molecules with low bioavailability (i.e., quercetin, ECGC, lycopene, and curcumin), sulforaphane exhibits a high bioavailability (80% compared to 4% for quercetin and 1% for curcumin) [173,174]. Thus, based on these promising findings, future studies should assess the clinical relevance of these organosulfur compounds in the treatment of PAD.

### 4.5. Dietary Interventions

In addition to testing individual phytochemicals, the Prevención con Dieta Mediterránea (PREDIMED) randomized trial assessed the effect of three different dietary interventions: a Mediterranean diet supplemented with olive oil, a Mediterranean diet supplemented with nuts, or a control low-fat diet [175,176]. At baseline, participants (*N* = 7447) aged 55 to 80 years had no clinical PAD or cardiovascular disease. In an exploratory analysis, both Mediterranean diet interventions were associated with a reduced PAD risk compared to the control diet at 5-year follow-up [177]. Furthermore, the reduction in PAD risk was greater in the group randomized to the Mediterranean diet supplemented with olive oil (hazard ratio of 0.34) compared to the Mediterranean diet supplemented with nuts (hazard ratio of 0.50), although there was no statistically significant difference between the two groups [177]. These results provide evidence for a relationship between diet and incident PAD. Consumption of a wide range of phytochemicals and antioxidants from fruits, vegetables, nuts, and olive oil may be beneficial for the prevention of PAD.

## 5. Conclusions

Phytochemicals have long been studied for their antioxidant activities, both as direct scavengers of ROS as well as by inhibition of pro-oxidant enzymes and upregulation of antioxidant enzymes [178]. More recently, phytochemicals have also been studied for the potential to counter endothelial dysfunction by increasing NO bioavailability [178]. While a large body of research has developed surrounding the cardioprotective effects of different phytochemicals, fewer studies are available in the context of PAD. The functional impairment characteristic of PAD is associated with increased oxidative stress and endothelial dysfunction [42]; thus, treatments targeting these mechanisms may be helpful in improving patients’ walking performance. Despite the promising findings of a number of preclinical studies using several phytochemical compounds, clinical trials in patients have been less successful. The poor bioavailability exhibited by several of the molecules discussed in the preclinical section may be the reason for significant laboratory potential but limited clinical usefulness. However, from the studies included in this review, it appears that beetroot and cocoa may be promising phytochemicals for improving functional status in PAD patients, with two studies for each compound demonstrating some degree of clinically significant improvements in walking performance. Since the compounds likely act via both shared and distinct mechanisms, it would be interesting to assess the utility of a combined beetroot + cocoa intervention. Furthermore, based on the findings of a number of preclinical studies as well as clinical trials in other diseases, organosulfur compounds may be promising therapeutic approaches for PAD treatment as well. Future studies should test sulforaphane as a potentially clinically relevant nutraceutical in the treatment of PAD. Additionally, none of the human studies assessed the effects on inflammatory molecules; since phytochemicals may have anti-inflammatory effects, future studies should also include inflammation markers as outcome measures. Another important point to consider is the potential long-term beneficial effects of these compounds, as the longest trial included in this review was only 6 months. Since data suggest a lack of durable PAD treatments [61], future studies should consider whether beneficial effects of phytochemicals can be sustained in the long-term. Finally, aside from the specific actions of certain phytochemicals, due to the broad health benefits of fruits and vegetables, consumption of a wide variety of fruits and vegetables (i.e., Mediterranean diet) should be recommended and encouraged for PAD patients.

## Figures and Tables

**Table 1:** Preclinical studies of phytochemicals in HLI models.

Compound	Dosage and Administration	Treatment Schedule	Species	HLI Induction	Results	Reference
Quercetin	200 mg/kg/day (orally)	1 week prior to HLI	Rat	Tourniquet	Reduced lipid peroxidation and increased antioxidant enzyme activity	[83]
	Glucosides: 100 mg/kg/day (gavage)	2 weeks prior to HLI and 2 weeks following	Mouse	Resection of femoral and saphenous artery	Enhanced blood flow recovery, increased capillary density	[85]
	185 mg/kg/day (orally)	30 days, beginning 5 days after HLI	Mouse	2-stages: 1st stage: ameroid constrictors around femoral artery; 2nd stage: resection of femoral artery	No effect on exercise performance or limb perfusion, but increased plasma nitric oxide metabolites	[84]
EGCG	25 or 50 mg/kg (i.p. injection)	30 min prior to reperfusion	Rat	Tourniquet (ischemia-reperfusion)	50 mg/kg reduced total oxidative status and increased total antioxidant status of skeletal muscle during acute reperfusion (2 h); neither dose affected oxidant/antioxidant status long-term (24 h); no effect on inflammatory cytokines	[87]
	Copper-EGCG (2 µg) (intramuscular injection)	1, 3, and 5 days after HLI	Mouse	Ligation and excision of femoral artery	Increased angiogenic markers, enhanced blood flow recovery, reduced ischemic damage, improved vessel volume	[91]
	Zinc-EGCG (0.682 µg) (intra-muscular injection)	1, 3, and 5 days after HLI	Mouse	Ligation and excision of femoral artery	Increased angiogenic markers, enhanced blood flow recovery, reduced ischemic damage, improved vessel volume	[90]
Catechin	1–10 mg/kg/day (orally)	5 days prior to HLI	Mouse	Ligation of femoral artery	Improved perfusion recovery and increased capillary density	[93]
Resveratrol	1 mg/kg/day (orally)	8 weeks following HLI	Rat	Tourniquet	Alleviated femoral artery tissue damage, reduced lipid peroxidation, and increased antioxidant expression	[111]
	10 mg/kg (i.p. injection)	Immediately prior to reperfusion	Rat	Tourniquet (ischemia-reperfusion)	Improved skeletal muscle histopathology, reduced lipid peroxidation	[112]
	20 mg/kg/day (orally)	2 weeks prior to HLI	Rat	Tourniquet (ischemia-reperfusion)	Reduced markers of muscle damage and oxidative stress markers	[113]
	2.5 mg/kg (loading subcutaneous dose), followed by 2.5 mg/kg/day (orally)	4 weeks following HLI	Rabbit	Resection of femoral, popliteal, and saphenous arteries, and ligation of external iliac artery	Increased limb blood pressure ratio, angiographic score, and angiogenic markers	[114]
	Intramuscular injection of bone marrow mononuclear cells incubated with 100 µM resveratrol	Immediately following HLI	Mouse	Ligation and excision of femoral artery	Enhanced blood flow recovery, increased capillary density and angiogenic markers, reduced ROS production	[115]
DATS	500 µg/kg/day (i.p. injection)	10 days following HLI	Mouse	Ligation of femoral artery	Enhanced blood flow recovery and capillary density, reduced oxidative stress	[120]
	500 µg/kg/day (i.p. injection)	2 weeks following HLI	Mouse	Ligation of femoral artery	Enhanced blood flow recovery and capillary density, increased angiogenic markers, increased NO reduced oxidative stress markers, increased tissue nitric oxide metabolites	[121]
Lycopene	10 mg/kg/day	15 days prior to HLI	Rat	Tourniquet (ischemia-reperfusion)	Reduced lipid peroxidation, increased antioxidant levels, improved skeletal muscle histopathology	[133]

**Table 2:** Clinical studies of phytochemicals in PAD patients.

Study (Ref.)	Compound	Dosage	Sample Size	Study Type	Intervention/ Intervention Length	Outcome Measure(s)	Difference or Change in Outcome Measure(s)	*p*-Value
Kenjale et al., 2011 (160)	treatment: beetroot juice placebo: orange juice	Nitrate content: 18,181 µmol/L	*N* = 8	randomized control cross-over trial	3 h (acute)	treadmill: claudication onset time, peak walking time; flow-mediated dilation	treadmill claudication onset time: placebo: 183 ± 84 s; beetroot: 215 ± 99 s treadmill peak walking time: placebo: 467 ± 223 s; beetroot: 533 ± 233 s	treadmill claudication onset time: *p* < 0.01 * treadmill peak walking time: *p* < 0.05 *
Woessner et al., 2018 (162)	Treatment: beetroot juice Placebo: nitrate-depleted identical drink	Nitrate content: 4.2 mmol	*N* = 24	randomized controlled trial	exercise (3×/wk) + daily beverage, 12 weeks	treadmill: claudication onset time; 6-min walking test: 6-min walking distance	treadmill claudication onset time: change in exercise + placebo group: 59.2 ± 57.3 s; change in exercise + beetroot group: 180.3 ± 46.6 s 6-min walking distance: change in exercise + placebo group: 24.6 ± 12.1 m; change in exercise + beetroot group: 53.4 ± 19.6 m	treadmill claudication onset time: *p* < 0.05 * 6-min walking distance: *p* < 0.05 *
Loffredo et al., 2014 (157)	Treatment: Cocoa Placebo: Milk chocolate	40 g dark chocolate (>85% cocoa)	*N* = 20	randomized control cross-over trial	2 h (acute)	treadmill: maximum walking distance, maximum walking time; flow-mediated dilation	treadmill maximum walking distance: placebo: 109.1 ± 65.1 m; Cocoa: 122.2 ± 61.5 treadmill maximum walking time: placebo: 125.4 ± 64.1 s; Cocoa: 142.2 ± 62.0 s flow-mediated dilation: placebo: 3.6 ± 3.2%; Cocoa: 6.3 ± 2.7%	treadmill maximum walking distance: *p* = 0.01 * treadmill maximum walking time: *p* = 0.006 * flow-mediated dilation: *p* = 0.003 *
Hammer et al., 2015 (156)	Treatment: Cocoa Placebo: Milk chocolate	50 g dark chocolate (70% cocoa content)	*N* = 21	randomized control cross-over trial	2 h (acute)	flow-mediated dilation	flow-mediated dilation: change in placebo group: −2.0%; change in Cocoa group: 0.4%	flow-mediated dilation: *p* = 0.18
McDermott et al., 2020 (158)	Treatment: Cocoa Placebo: Identical, cocoa-free beverage	15 g cocoa (75 mg epicatechin)	*N* = 44	randomized control trial	Daily beverage, 6 months	6-min walking test: 6-min walking distance 2.5 h, 6-min walking distance 24 h, treadmill: pain-free walking time, maximum walking time; flow-mediated dilation 2.5 h; flow-mediated dilation 24 h	6-min walking distance 2.5 h: change in placebo group: from 337.3 ± 85.2 m to 322.0 ± 96.4 m; change in Cocoa group: from 348.6 ± 74.2 m to 356.6 ± 64.0 m 6-min walking distance 24 h: change in placebo group: from 329.1 ± 90.4 m to 335.4 ± 92.5 m; change in Cocoa group: from 347.7 ± 74.3 m to 353.0 ± 76.9 m	6-min walking distance 2.5 h: *p* = 0.005 * 6-min walking distance 24 h: *p* = 0.12 flow-mediated dilation: 2.5 h: *p* = 0.84; 24 h: *p* = 0.30 treadmill pain-free walking time: *p* = 0.68 treadmill maximum walking time: *p* = 0.62
Kieswetter et al., 1993 (166)	Treatment: Garlic Placebo: coated cellulose tablets	Garlic coated tablets (800 mg)	*N* = 80	randomized control trial	physical therapy (2×/wk) + daily supplement, 12 weeks	treadmill: pain-free walking distance	treadmill pain-free walking distance: change in placebo group: 172 ± 60.9 m to 203.1 ± 72.8 m change in Garlic group: from 161 ± 65.1 m to 207.1 ± 85 m	treadmill pain-free walking distance: *p* < 0.038 *
McDermott et al., 2017 (165)	Treatment: Resveratrol Placebo: Placebo pill	125 mg/day or 500 mg/day	*N* = 66	randomized control trial	Daily supplement, 6 months	6-min walking test: 6-min walking distance; treadmill: maximum walking time	6-min walking distance: change in placebo group: −12.3 ± 7.9 m; change in 125 mg group: 4.6 ± 8.1 m; change in 500 mg group: −12.8 ± 7.5 m treadmill maximum walking time: change in placebo group: 0.4 ± 2.1 min; change in 125 mg group: 0.5 ± 2.3 min; change in 500 mg group: −0.6 ± 2.1 min	6-min walking distance: 125 mg vs. placebo: *p* = 0.07 *; 500 mg vs. placebo: *p* = 0.96 treadmill maximum walking time: 125 mg vs. placebo: *p* = 0.18; 500 mg vs. placebo: *p* = 0.12

Note: * indicates significant difference, as defined by study.

## References

[1] Mozaffarian D., Rosenberg I., Uauy R. (2018). History of modern nutrition science-implications for current research, dietary guidelines, and food policy. BMJ.

[2] Staub P.O., Casu L., Leonti M. (2016). Back to the roots: A quantitative survey of herbal drugs in Dioscorides’ De Materia Medica (ex Matthioli, 1568). Phytomedicine.

[3] Carpenter K.J. (2003). A short history of nutritional science: Part 4 (1945–1985). J. Nutr..

[4] Wang X., Ouyang Y.Y., Liu J., Zhu M.M., Zhao G., Bao W., Hu F.B. (2014). Fruit and vegetable consumption and mortality from all causes, cardiovascular disease, and cancer: Systematic review and dose-response meta-analysis of prospective cohort studies. BMJ.

[5] Bellavia A., Larsson S.C., Bottai M., Wolk A., Orsini N. (2013). Fruit and vegetable consumption and all-cause mortality: A dose-response analysis. Am. J. Clin. Nutr..

[6] Hung H.C., Joshipura K.J., Jiang R., Hu F.B., Hunter D., Smith-Warner S.A., Colditz G.A., Rosner B., Spiegelman D., Willett W.C. (2004). Fruit and vegetable intake and risk of major chronic disease. JNCI.

[7] Aune D., Giovannucci E., Boffetta P., Fadnes L.T., Keum N., Norat T., Greenwood D.C., Riboli E., Vatten L.J., Tonstad S. (2017). Fruit and vegetable intake and the risk of cardiovascular disease, total cancer and all-cause mortality-a systematic review and dose-response meta-analysis of prospective studies. Int. J. Epidemiol..

[8] Momiyama Y., Adachi H., Fairweather D., Ishizaka N., Saita E. (2014). Inflammation, Atherosclerosis and Coronary Artery Disease. Clin. Med. Insights Cardiol..

[9] Blekkenhorst L.C., Bondonno C.P., Lewis J.R., Woodman R.J., Devine A., Bondonno N.P., Lim W.H., Zhu K., Beilin L.J., Thompson P.L. (2018). Cruciferous and Total Vegetable Intakes Are Inversely Associated with Subclinical Atherosclerosis in Older Adult Women. J. Am. Heart Assoc..

[10] Guo W., Kim S.H., Wu D., Li L., Ortega E.F., Thomas M., Meydani S.N., Meydani M. (2021). Dietary Fruit and Vegetable Supplementation Suppresses Diet-Induced Atherosclerosis in LDL Receptor Knockout Mice. J. Nutr..

[11] Kullo I.J., Rooke T.W. (2016). CLINICAL PRACTICE. Peripheral Artery Disease. N. Engl. J. Med..

[12] Chen Q., Smith C.Y., Bailey K.R., Wennberg P.W., Kullo I.J. (2013). Disease Location Is Associated with Survival in Patients with Peripheral Arterial Disease. J. Am. Heart Assoc..

[13] Heffron S.P., Rockman C.B., Adelman M.A., Gianos E., Guo Y., Xu J.F., Berger J.S. (2017). Greater Frequency of Fruit and Vegetable Consumption Is Associated with Lower Prevalence of Peripheral Artery Disease. Arterioscl. Throm. Vas..

[14] Ciccarone E., Di Castelnuovo A., Salcuni M., Siani A., Giacco A., Donati M.B., De Gaetano G., Capani F., Iacoviello L., Inv G. (2003). A high-score Mediterranean dietary pattern is associated with a reduced risk of peripheral arterial disease in Italian patients with Type 2 diabetes. J. Thromb. Haemost..

[15] Howard B.V., Kritchevsky D. (1997). Phytochemicals and cardiovascular disease. A statement for healthcare professionals from the American Heart Association. Circulation.

[16] Chen H., Liu R.H. (2018). Potential Mechanisms of Action of Dietary Phytochemicals for Cancer Prevention by Targeting Cellular Signaling Transduction Pathways. J. Agric. Food Chem..

[17] Roleira F.M., Tavares-da-Silva E.J., Varela C.L., Costa S.C., Silva T., Garrido J., Borges F. (2015). Plant derived and dietary phenolic antioxidants: Anticancer properties. Food Chem..

[18] Owens D.J., Twist C., Cobley J.N., Howatson G., Close G.L. (2019). Exercise-induced muscle damage: What is it, what causes it and what are the nutritional solutions?. Eur. J. Sport Sci..

[19] Itoh K., Chiba T., Takahashi S., Ishii T., Igarashi K., Katoh Y., Oyake T., Hayashi N., Satoh K., Hatayama I. (1997). An Nrf2/small Maf heterodimer mediates the induction of phase II detoxifying enzyme genes through antioxidant response elements. Biochem. Biophys. Res. Commun..

[20] Fukunaga B.N., Probst M.R., Reisz-Porszasz S., Hankinson O. (1995). Identification of functional domains of the aryl hydrocarbon receptor. J. Biol. Chem..

[21] Gu X., Zhu Y.Z. (2011). Therapeutic applications of organosulfur compounds as novel hydrogen sulfide donors and/or mediators. Expert Rev. Clin. Pharmacol..

[22] Altaany Z., Yang G., Wang R. (2013). Crosstalk between hydrogen sulfide and nitric oxide in endothelial cells. J. Cell Mol. Med..

[23] Corsello T., Komaravelli N., Casola A. (2018). Role of Hydrogen Sulfide in NRF2- and Sirtuin-Dependent Maintenance of Cellular Redox Balance. Antioxidants.

[24] Ismaeel A., Papoutsi E., Miserlis D., Lavado R., Haynatzki G., Casale G.P., Bohannon W.T., Smith R.S., Eidson J.L., Brumberg R. (2020). The Nitric Oxide System in Peripheral Artery Disease: Connection with Oxidative Stress and Biopterins. Antioxidants.

[25] Islam K.N., Polhemus D.J., Donnarumma E., Brewster L.P., Lefer D.J. (2015). Hydrogen Sulfide Levels and Nuclear Factor-Erythroid 2-Related Factor 2 (NRF2) Activity Are Attenuated in the Setting of Critical Limb Ischemia (CLI). J. Am. Heart Assoc..

[26] Ranjan A., Ramachandran S., Gupta N., Kaushik I., Wright S., Srivastava S., Das H., Srivastava S., Prasad S., Srivastava S.K. (2019). Role of Phytochemicals in Cancer Prevention. Int. J. Mol. Sci..

[27] Di Meglio F., Sacco A., Belviso I., Romano V., Sirico F., Loiacono C., Palermi S., Pempinello C., Montagnani S., Nurzynska D. (2020). Influence of Supplements and Drugs used for the Treatment of Musculoskeletal Disorders on Adult Human Tendon-Derived Stem Cells. Muscles Ligaments Tendons J. (MLTJ).

[28] Pagliaro B., Santolamazza C., Simonelli F., Rubattu S. (2015). Phytochemical Compounds and Protection from Cardiovascular Diseases: A State of the Art. Biomed. Res. Int..

[29] Kumar G.P., Khanum F. (2012). Neuroprotective potential of phytochemicals. Pharmacogn. Rev..

[30] Fowkes F.G., Rudan D., Rudan I., Aboyans V., Denenberg J.O., McDermott M.M., Norman P.E., Sampson U.K., Williams L.J., Mensah G.A. (2013). Comparison of global estimates of prevalence and risk factors for peripheral artery disease in 2000 and 2010: A systematic review and analysis. Lancet.

[31] Mortality G.B.D., Causes of Death C. (2016). Global, regional, and national life expectancy, all-cause mortality, and cause-specific mortality for 249 causes of death, 1980-2015: A systematic analysis for the Global Burden of Disease Study 2015. Lancet.

[32] Savji N., Rockman C.B., Skolnick A.H., Guo Y., Adelman M.A., Riles T., Berger J.S. (2013). Association Between Advanced Age and Vascular Disease in Different Arterial Territories A Population Database of Over 3.6 Million Subjects. J. Am. Coll. Cardiol..

[33] Norgren L., Hiatt W.R., Dormandy J.A., Nehler M.R., Harris K.A., Fowkes F.G., Group T.I.W., Bell K., Caporusso J., Durand-Zaleski I. (2007). Inter-Society Consensus for the Management of Peripheral Arterial Disease (TASC II). Eur. J. Vasc. Endovasc. Surg..

[34] Criqui M.H., Langer R.D., Fronek A., Feigelson H.S., Klauber M.R., McCann T.J., Browner D. (1992). Mortality over a period of 10 years in patients with peripheral arterial disease. N. Engl. J. Med..

[35] Caro J., Migliaccio-Walle K., Ishak K.J., Proskorovsky I. (2005). The morbidity and mortality following a diagnosis of peripheral arterial disease: Long-term follow-up of a large database. BMC Cardiovasc. Disord.

[36] Steg P.G., Bhatt D.L., Wilson P.W., D’Agostino R., Ohman E.M., Rother J., Liau C.S., Hirsch A.T., Mas J.L., Ikeda Y. (2007). One-year cardiovascular event rates in outpatients with atherothrombosis. JAMA.

[37] Gardner A.W. (1993). Claudication pain and hemodynamic responses to exercise in younger and older peripheral arterial disease patients. J. Gerontol..

[38] Gardner A.W., Killewich L.A., Katzel L.I., Womack C.J., Montgomery P.S., Otis R.B., Fonong T. (1999). Relationship between free-living daily physical activity and peripheral circulation in patients with intermittent claudication. Angiology.

[39] Hiatt W.R., Armstrong E.J., Larson C.J., Brass E.P. (2015). Pathogenesis of the limb manifestations and exercise limitations in peripheral artery disease. Circ. Res..

[40] Koutakis P., Ismaeel A., Farmer P., Purcell S., Smith R.S., Eidson J.L., Bohannon W.T. (2018). Oxidative stress and antioxidant treatment in patients with peripheral artery disease. Physiol. Rep..

[41] McDermott M.M., Ferrucci L., Gonzalez-Freire M., Kosmac K., Leeuwenburgh C., Peterson C.A., Saini S., Sufit R. (2020). Skeletal Muscle Pathology in Peripheral Artery Disease: A Brief Review. Arterioscler. Thromb. Vasc. Biol..

[42] Ismaeel A., Brumberg R.S., Kirk J.S., Papoutsi E., Farmer P.J., Bohannon W.T., Smith R.S., Eidson J.L., Sawicki I., Koutakis P. (2018). Oxidative Stress and Arterial Dysfunction in Peripheral Artery Disease. Antioxidants.

[43] Dopheide J.F., Doppler C., Scheer M., Obst V., Radmacher M.C., Radsak M.P., Gori T., Warnholtz A., Fottner C., Munzel T. (2013). Critical limb ischaemia is characterised by an increased production of whole blood reactive oxygen species and expression of TREM-1 on neutrophils. Atherosclerosis.

[44] Weiss D.J., Casale G.P., Koutakis P., Nella A.A., Swanson S.A., Zhu Z., Miserlis D., Johanning J.M., Pipinos I.I. (2013). Oxidative damage and myofiber degeneration in the gastrocnemius of patients with peripheral arterial disease. J. Transl. Med..

[45] Kraiss L., Chong K. (2014). Walking disability in patients with peripheral artery disease is associated with arterial endothelial function DISCUSSION. J. Vasc Surg.

[46] Brevetti G., Giugliano G., Brevetti L., Hiatt W.R. (2010). Inflammation in peripheral artery disease. Circulation.

[47] Ridker P.M., Cushman M., Stampfer M.J., Tracy R.P., Hennekens C.H. (1998). Plasma concentration of C-reactive protein and risk of developing peripheral vascular disease. Circulation.

[48] Tzoulaki I., Murray G.D., Lee A.J., Rumley A., Lowe G.D., Fowkes F.G. (2007). Inflammatory, haemostatic, and rheological markers for incident peripheral arterial disease: Edinburgh Artery Study. Eur. Heart J..

[49] Ross R. (1999). Atherosclerosis--an inflammatory disease. N. Engl. J. Med..

[50] Zanoli L., Rastelli S., Inserra G., Castellino P. (2015). Arterial structure and function in inflammatory bowel disease. World J. Gastroenterol..

[51] Weber C., Zernecke A., Libby P. (2008). The multifaceted contributions of leukocyte subsets to atherosclerosis: Lessons from mouse models. Nat. Rev. Immunol..

[52] Jialal I., Verma S., Devaraj S. (2009). Inhibition of endothelial nitric oxide synthase by C-reactive protein: Clinical relevance. Clin. Chem..

[53] Barbato J.E., Tzeng E. (2004). Nitric oxide and arterial disease. J. Vasc. Surg..

[54] McDermott M.M., Liu K., Greenland P., Guralnik J.M., Criqui M.H., Chan C., Pearce W.H., Schneider J.R., Ferrucci L., Celic L. (2004). Functional decline in peripheral arterial disease: Associations with the ankle brachial index and leg symptoms. JAMA.

[55] McDermott M.M., Guralnik J.M., Criqui M.H., Liu K., Kibbe M.R., Ferrucci L. (2014). Six-Minute Walk Is a Better Outcome Measure Than Treadmill Walking Tests in Therapeutic Trials of Patients With Peripheral Artery Disease. Circulation.

[56] McDermott M.M., Tian L., Criqui M.H., Ferrucci L., Conte M.S., Zhao L., Li L., Sufit R., Polonsky T.S., Kibbe M.R. (2021). Meaningful change in 6-min walk in people with peripheral artery disease. J. Vasc. Surg..

[57] Gardner A.W., Montgomery P.S., Wang M. (2018). Minimal clinically important differences in treadmill, 6-min walk, and patient-based outcomes following supervised and home-based exercise in peripheral artery disease. Vasc. Med..

[58] Salhiyyah K., Forster R., Senanayake E., Abdel-Hadi M., Booth A., Michaels J.A. (2015). Pentoxifylline for intermittent claudication. Cochrane Database Syst. Rev..

[59] Dawson D.L., Cutler B.S., Hiatt W.R., Hobson R.W., Martin J.D., Bortey E.B., Forbes W.P., Strandness D.E. (2000). A comparison of cilostazol and pentoxifylline for treating intermittent claudication. Am. J. Med..

[60] Treat-Jacobson D., McDermott M.M., Bronas U.G., Campia U., Collins T.C., Criqui M.H., Gardner A.W., Hiatt W.R., Regensteiner J.G., Rich K. (2019). Optimal Exercise Programs for Patients with Peripheral Artery Disease: A Scientific Statement From the American Heart Association. Circulation.

[61] Thanigaimani S., Phie J., Sharma C., Wong S., Ibrahim M., Huynh P., Moxon J., Jones R., Golledge J. (2021). Network Meta-Analysis Comparing the Outcomes of Treatments for Intermittent Claudication Tested in Randomized Controlled Trials. J. Am. Heart Assoc..

[62] Li S., Myers S.A., Thompson J., Kim J., Koutakis P., Williams M., Zhu Z., Schieber M., Lackner T., Willcockson G. (2020). Different Outcomes after Revascularization or Standard Supervised Exercise Treadmill Training of Claudicating Patients with Peripheral Artery Disease. JVS-Vasc. Sci..

[63] Hiatt W.R., Regensteiner J.G., Wolfel E.E., Carry M.R., Brass E.P. (1996). Effect of exercise training on skeletal muscle histology and metabolism in peripheral arterial disease. J. Appl. Physiol..

[64] Hickman P., Harrison D.K., Hill A., McLaren M., Tamei H., McCollum P.T., Belch J.J. (1994). Exercise in patients with intermittent claudication results in the generation of oxygen derived free radicals and endothelial damage. Adv. Exp. Med. Biol..

[65] Silvestro A., Scopacasa F., Oliva G., de Cristofaro T., Iuliano L., Brevetti G. (2002). Vitamin C prevents endothelial dysfunction induced by acute exercise in patients with intermittent claudication. Atherosclerosis.

[66] Hamburg N.M., Balady G.J. (2011). Exercise rehabilitation in peripheral artery disease: Functional impact and mechanisms of benefits. Circulation.

[67] Belch J., MacCuish A., Campbell I., Cobbe S., Taylor R., Prescott R., Lee R., Bancroft J., MacEwan S., Shepherd J. (2008). The prevention of progression of arterial disease and diabetes (POPADAD) trial: Factorial randomised placebo controlled trial of aspirin and antioxidants in patients with diabetes and asymptomatic peripheral arterial disease. BMJ.

[68] Lonn E., Yusuf S., Hoogwerf B., Pogue J., Yi Q., Zinman B., Bosch J., Dagenais G., Mann J.F., Gerstein H.C. (2002). Effects of vitamin E on cardiovascular and microvascular outcomes in high-risk patients with diabetes: Results of the HOPE study and MICRO-HOPE substudy. Diabetes Care.

[69] Cobley J.N., McHardy H., Morton J.P., Nikolaidis M.G., Close G.L. (2015). Influence of vitamin C and vitamin E on redox signaling: Implications for exercise adaptations. Free Radic. Biol. Med..

[70] Drummond G.R., Selemidis S., Griendling K.K., Sobey C.G. (2011). Combating oxidative stress in vascular disease: NADPH oxidases as therapeutic targets. Nat. Rev. Drug. Discov..

[71] Couffinhal T., Silver M., Zheng L.P., Kearney M., Witzenbichler B., Isner J.M. (1998). Mouse model of angiogenesis. Am. J. Pathol..

[72] Krishna S.M., Omer S.M., Golledge J. (2016). Evaluation of the clinical relevance and limitations of current pre-clinical models of peripheral artery disease. Clin. Sci..

[73] Niiyama H., Huang N.F., Rollins M.D., Cooke J.P. (2009). Murine model of hindlimb ischemia. J. Vis. Exp..

[74] Padgett M.E., McCord T.J., McClung J.M., Kontos C.D. (2016). Methods for Acute and Subacute Murine Hindlimb Ischemia. J. Vis. Exp..

[75] Pipinos I.I., Swanson S.A., Zhu Z., Nella A.A., Weiss D.J., Gutti T.L., McComb R.D., Baxter B.T., Lynch T.G., Casale G.P. (2008). Chronically ischemic mouse skeletal muscle exhibits myopathy in association with mitochondrial dysfunction and oxidative damage. Am. J. Physiol. Regul. Integr. Comp. Physiol..

[76] Krishna S.M., Omer S.M., Li J., Morton S.K., Jose R.J., Golledge J. (2020). Development of a two-stage limb ischemia model to better simulate human peripheral artery disease. Sci. Rep..

[77] Thanigaimani S., Phie J., Golledge J. (2020). Animal models of ischemic limb ulcers: A systematic review and meta-analysis. BMJ Open Diabetes Res. Care.

[78] Kwik-Uribe C., Bektash R.M. (2008). Cocoa flavanols-Measurement, bioavailability and bioactivity. Asia. Pac. J. Clin. Nutr..

[79] Panche A.N., Diwan A.D., Chandra S.R. (2016). Flavonoids: An overview. J. Nutr. Sci..

[80] Phie J., Krishna S.M., Moxon J.V., Omer S.M., Kinobe R., Golledge J. (2017). Flavonols reduce aortic atherosclerosis lesion area in apolipoprotein E deficient mice: A systematic review and meta-analysis. PLoS ONE.

[81] Manach C., Scalbert A., Morand C., Remesy C., Jimenez L. (2004). Polyphenols: Food sources and bioavailability. Am. J. Clin. Nutr..

[82] Williams R.J., Spencer J.P., Rice-Evans C. (2004). Flavonoids: Antioxidants or signalling molecules?. Free Radic. Biol. Med..

[83] Ekinci Akdemir F.N., Gulcin I., Karagoz B., Soslu R. (2016). Quercetin protects rat skeletal muscle from ischemia reperfusion injury. J. Enzyme Inhib. Med. Chem..

[84] Sumi M., Tateishi N., Shibata H., Ohki T., Sata M. (2013). Quercetin glucosides promote ischemia-induced angiogenesis, but do not promote tumor growth. Life Sci..

[85] Phie J., Krishna S.M., Kinobe R., Moxon J.V., Andrade-Lima A., Morton S.K., Lazzaroni S.M., Huynh P., Golledge J. (2021). Effects of quercetin on exercise performance, physical activity and blood supply in a novel model of sustained hind-limb ischaemia. BJS Open.

[86] Lorenz M., Urban J., Engelhardt U., Baumann G., Stangl K., Stangl V. (2009). Green and black tea are equally potent stimuli of NO production and vasodilation: New insights into tea ingredients involved. Basic Res. Cardiol..

[87] Ergun Y., Kilinc M., Aral M., Hedef A., Kaya E. (2020). Protective Effect of Epigallocatechin Gallate in Ischemia-Reperfusion Injury of Rat Skeletal Muscle. J. Surg. Res..

[88] Rahim M.A., Ejima H., Cho K.L., Kempe K., Mullner M., Best J.P., Caruso F. (2014). Coordination-Driven Multistep Assembly of Metal-Polyphenol Films and Capsules. Chem. Mater..

[89] Ejima H., Richardson J.J., Liang K., Best J.P., van Koeverden M.P., Such G.K., Cui J.W., Caruso F. (2013). One-Step Assembly of Coordination Complexes for Versatile Film and Particle Engineering. Science.

[90] Chen Z., Duan J., Diao Y., Chen Y., Liang X., Li H., Miao Y., Gao Q., Gui L., Wang X. (2021). ROS-responsive capsules engineered from EGCG-Zinc networks improve therapeutic angiogenesis in mouse limb ischemia. Bioact. Mater..

[91] Duan J., Chen Z., Liang X., Chen Y., Li H., Tian X., Zhang M., Wang X., Sun H., Kong D. (2020). Construction and application of therapeutic metal-polyphenol capsule for peripheral artery disease. Biomaterials.

[92] Carnevale R., Loffredo L., Nocella C., Bartimoccia S., Bucci T., De Falco E., Peruzzi M., Chimenti I., Biondi-Zoccai G., Pignatelli P. (2014). Epicatechin and catechin modulate endothelial activation induced by platelets of patients with peripheral artery disease. Oxid. Med. Cell Longev..

[93] Schuler D. (2013). (−)-Epicatechin increases angiogenesis after hindlimb ischemia. Nitric. Oxide Biol. Ch..

[94] Lidder S., Webb A.J. (2013). Vascular effects of dietary nitrate (as found in green leafy vegetables and beetroot) via the nitrate-nitrite-nitric oxide pathway. Br. J. Clin. Pharmacol..

[95] Hendgen-Cotta U.B., Luedike P., Totzeck M., Kropp M., Schicho A., Stock P., Rammos C., Niessen M., Heiss C., Lundberg J.O. (2012). Dietary nitrate supplementation improves revascularization in chronic ischemia. Circulation.

[96] Kumar D., Branch B.G., Pattillo C.B., Hood J., Thoma S., Simpson S., Illum S., Arora N., Chidlow J.H., Langston W. (2008). Chronic sodium nitrite therapy augments ischemia-induced angiogenesis and arteriogenesis. Proc. Natl. Acad. Sci. USA.

[97] Bradley J.M., Islam K.N., Polhemus D.J., Donnarumma E., Brewster L.P., Tao Y.X., Goodchild T.T., Lefer D.J. (2015). Sustained release nitrite therapy results in myocardial protection in a porcine model of metabolic syndrome with peripheral vascular disease. Am. J. Physiol Heart Circ. Physiol..

[98] Polhemus D.J., Bradley J.M., Islam K.N., Brewster L.P., Calvert J.W., Tao Y.X., Chang C.C., Pipinos I.I., Goodchild T.T., Lefer D.J. (2015). Therapeutic potential of sustained-release sodium nitrite for critical limb ischemia in the setting of metabolic syndrome. Am. J. Physiol. Heart Circ. Physiol..

[99] Stafford H.A. (1994). Anthocyanins and Betalains-Evolution of the Mutually Exclusive Pathways. Plant. Sci..

[100] Hubbard T.D., Murray I.A., Perdew G.H. (2015). Indole and Tryptophan Metabolism: Endogenous and Dietary Routes to Ah Receptor Activation. Drug. Metab. Dispos..

[101] Tesoriere L., Allegra M., Butera D., Livrea M.A. (2004). Absorption, excretion, and distribution of dietary antioxidant betalains in LDLs: Potential health effects of betalains in humans. Am. J. Clin. Nutr..

[102] Vulic J.J., Cebovic T.N., Canadanovic-Brunet J.M., Cetkovic G.S., Canadanovic V.M., Djilas S.M., Saponjac V.T.T. (2014). In vivo and in vitro antioxidant effects of beetroot pomace extracts. J. Funct. Foods.

[103] Vidal P.J., Lopez-Nicolas J.M., Gandia-Herrero F., Garcia-Carmona F. (2014). Inactivation of lipoxygenase and cyclooxygenase by natural betalains and semi-synthetic analogues. Food Chem..

[104] Traverse J.H. (2018). “Beet It”. Circ. Res..

[105] Salloum F.N., Sturz G.R., Yin C., Rehman S., Hoke N.N., Kukreja R.C., Xi L. (2015). Beetroot juice reduces infarct size and improves cardiac function following ischemia-reperfusion injury: Possible involvement of endogenous H2S. Exp. Biol. Med..

[106] Akinwumi B.C., Bordun K.M., Anderson H.D. (2018). Biological Activities of Stilbenoids. Int. J. Mol. Sci..

[107] Baur J.A., Pearson K.J., Price N.L., Jamieson H.A., Lerin C., Kalra A., Prabhu V.V., Allard J.S., Lopez-Lluch G., Lewis K. (2006). Resveratrol improves health and survival of mice on a high-calorie diet. Nature.

[108] Howitz K.T., Bitterman K.J., Cohen H.Y., Lamming D.W., Lavu S., Wood J.G., Zipkin R.E., Chung P., Kisielewski A., Zhang L.L. (2003). Small molecule activators of sirtuins extend Saccharomyces cerevisiae lifespan. Nature.

[109] Milne J.C., Lambert P.D., Schenk S., Carney D.P., Smith J.J., Gagne D.J., Jin L., Boss O., Perni R.B., Vu C.B. (2007). Small molecule activators of SIRT1 as therapeutics for the treatment of type 2 diabetes. Nature.

[110] Baur J.A., Sinclair D.A. (2006). Therapeutic potential of resveratrol: The in vivo evidence. Nat. Rev. Drug. Discov..

[111] Song X., Liu Z., Zeng R., Shao J., Zheng Y., Ye W. (2021). Resveratrol Alleviates Vascular Endothelial Damage Caused by Lower-Extremity Ischemia Reperfusion (I/R) through Regulating Keap1/Nrf2 Signaling-Mediated Oxidative Stress. Evid. Based Complement. Alternat. Med..

[112] Elmali N., Esenkaya I., Karadag N., Tas F., Elmali N. (2007). Effects of resveratrol on skeletal muscle in ischemia-reperfusion injury. Ulus. Travma Acil Cerrahi Derg..

[113] Ikizler M., Ovali C., Dernek S., Erkasap N., Sevin B., Kaygisiz Z., Kural T. (2006). Protective effects of resveratrol in ischemia-reperfusion injury of skeletal muscle: A clinically relevant animal model for lower extremity ischemia. Chin. J. Physiol..

[114] El Eter E.A., Al Zamil H., Deif M. (2013). Enhancement of Collaterals by Resveratrol in a Rabbit Model of Hind Limb Ischemia. Circulation.

[115] Gan L., Matsuura H., Ichiki T., Yin X., Miyazaki R., Hashimoto T., Cui J., Takeda K., Sunagawa K. (2009). Improvement of neovascularization capacity of bone marrow mononuclear cells from diabetic mice by ex vivo pretreatment with resveratrol. Hypertens Res..

[116] El-Awady M.H., El-Ghetany H.H., Aboelghait K.M., Dahaba A.A. (2019). Bioactivities of Allicin and Related Organosulfur Compounds from Garlic: Overview of the Literature Since 2010. Egypt J. Chem..

[117] Gunther W.H.H. (2013). Garlic and other alliums-The lore and the science. J. Sulfur. Chem..

[118] Chiang Y.H., Jen L.N., Su H.Y., Lii C.K., Sheen L.Y., Liu C.T. (2006). Effects of garlic oil and two of its major organosulfur compounds, diallyl disulfide and diallyl trisulfide, on intestinal damage in rats injected with endotoxin. Toxicol. Appl. Pharmacol..

[119] Predmore B.L., Kondo K., Bhushan S., Zlatopolsky M.A., King A.L., Aragon J.P., Grinsfelder D.B., Condit M.E., Lefer D.J. (2012). The polysulfide diallyl trisulfide protects the ischemic myocardium by preservation of endogenous hydrogen sulfide and increasing nitric oxide bioavailability. Am. J. Physiol. Heart Circ. Physiol..

[120] Hayashida R., Kondo K., Morita S., Unno K., Shintani S., Shimizu Y., Calvert J.W., Shibata R., Murohara T. (2017). Diallyl Trisulfide Augments Ischemia-Induced Angiogenesis via an Endothelial Nitric Oxide Synthase-Dependent Mechanism. Circ. J..

[121] Yang H.B., Liu H.M., Yan J.C., Lu Z.Y. (2018). Effect of Diallyl Trisulfide on Ischemic Tissue Injury and Revascularization in a Diabetic Mouse Model. J. Cardiovasc. Pharmacol..

[122] Ma Q. (2013). Role of nrf2 in oxidative stress and toxicity. Annu. Rev. Pharmacol. Toxicol..

[123] Yu C., He Q., Zheng J., Li L.Y., Hou Y.H., Song F.Z. (2017). Sulforaphane improves outcomes and slows cerebral ischemic/reperfusion injury via inhibition of NLRP3 inflammasome activation in rats. Int. Immunopharmacol..

[124] Ambrecht L., McDonnell J.F., Perlman J.I., Bu P. (2014). Protected Retinal Function by Sulforaphane on Retinal Ischemic Injury. Invest. Ophth. Vis. Sci..

[125] Chen Z.Q., Mohr A., Heitplatz B., Hansen U., Pascher A., Brockmann J.G., Becker F. (2020). Sulforaphane Elicits Protective Effects in Intestinal Ischemia Reperfusion Injury. Int. J. Mol. Sci..

[126] Silva-Palacios A., Ostolga-Chavarria M., Sanchez-Garibay C., Rojas-Morales P., Galvan-Arzate S., Buelna-Chontal M., Pavon N., Pedraza-Chaverri J., Konigsberg M., Zazueta C. (2019). Sulforaphane protects from myocardial ischemia-reperfusion damage through the balanced activation of Nrf2/AhR. Free Radical. Biol. Med..

[127] Shokeir A.A., Barakat N., Hussein A.M., Awadalla A., Harraz A.M., Khater S., Hemmaid K., Kamal A.I. (2015). Activation of Nrf2 by Ischemic Preconditioning and Sulforaphane in Renal Ischemia/Reperfusion Injury: A Comparative Experimental Study. Physiol. Res..

[128] Bose C., Alves I., Singh P., Palade P.T., Carvalho E., Borsheim E., Jun S.R., Cheema A., Boerma M., Awasthi S. (2020). Sulforaphane prevents age-associated cardiac and muscular dysfunction through Nrf2 signaling. Aging Cell.

[129] Shehatou G.S., Suddek G.M. (2016). Sulforaphane attenuates the development of atherosclerosis and improves endothelial dysfunction in hypercholesterolemic rabbits. Exp. Biol. Med..

[130] Britton G. (1995). Structure and properties of carotenoids in relation to function. FASEB J..

[131] Donnan P.T., Thomson M., Fowkes F.G.R., Prescott R.J., Housley E. (1993). Diet as a Risk Factor for Peripheral Arterial-Disease in the General-Population—The Edinburgh-Artery-Study. Am. J. Clin. Nutr..

[132] Klipstein-Grobusch K., den Breeijen J.H., Grobbee D.E., Boeing H., Hofman A., Witteman J.C. (2001). Dietary antioxidants and peripheral arterial disease: The Rotterdam Study. Am. J. Epidemiol..

[133] Kirisci M., Guneri B., Seyithanoglu M., Kazanci U., Doganer A., Gunes H. (2020). The protective effects of lycopene on ischemia/reperfusion injury in rat hind limb muscle model. Ulus. Travma Acil Cerrahi Derg..

[134] Li X., Jia P., Huang Z., Liu S., Miao J., Guo Y., Wu N., Jia D. (2019). Lycopene protects against myocardial ischemia-reperfusion injury by inhibiting mitochondrial permeability transition pore opening. Drug. Des. Devel. Ther..

[135] Hsiao G., Fong T.H., Tzu N.H., Lin K.H., Chou D.S., Sheu J.R. (2004). A potent antioxidant, lycopene, affords neuroprotection against microglia activation and focal cerebral ischemia in rats. In Vivo.

[136] Borel P., Desmarchelier C., Dumont U., Halimi C., Lairon D., Page D., Sebedio J.L., Buisson C., Buffiere C., Remond D. (2016). Dietary calcium impairs tomato lycopene bioavailability in healthy humans. Br. J. Nutr..

[137] Moran N.E., Clinton S.K., Erdman J.W. (2013). Differential bioavailability, clearance, and tissue distribution of the acyclic tomato carotenoids lycopene and phytoene in mongolian gerbils. J. Nutr..

[138] Collins J.K., Arjmandi B.H., Claypool P.L., Perkins-Veazie P., Baker R.A., Clevidence B.A. (2004). Lycopene from two food sources does not affect antioxidant or cholesterol status of middle-aged adults. Nutr. J..

[139] Cikrikci S., Mozioglu E., Yilmaz H. (2008). Biological Activity of Curcuminoids Isolated from Curcuma longa. Rec. Nat. Prod..

[140] Liu Y., Chen L.Y., Shen Y., Tan T., Xie N.Z., Luo M., Li Z.H., Xie X.Y. (2016). Curcumin Ameliorates Ischemia-Induced Limb Injury Through Immunomodulation. Med. Sci. Monitor.

[141] Zhang J., Wang Q., Rao G., Qiu J., He R. (2019). Curcumin improves perfusion recovery in experimental peripheral arterial disease by upregulating microRNA-93 expression. Exp. Ther. Med..

[142] You J., Sun J., Ma T., Yang Z., Wang X., Zhang Z., Li J., Wang L., Ii M., Yang J. (2017). Curcumin induces therapeutic angiogenesis in a diabetic mouse hindlimb ischemia model via modulating the function of endothelial progenitor cells. Stem. Cell Res. Ther..

[143] Nelson K.M., Dahlin J.L., Bisson J., Graham J., Pauli G.F., Walters M.A. (2017). The Essential Medicinal Chemistry of Curcumin. J. Med. Chem..

[144] Baker M. (2017). Deceptive curcumin offers cautionary tale for chemists. Nature.

[145] Karas M., Jakubczyk A., Szymanowska U., Zlotek U., Zielinska E. (2017). Digestion and bioavailability of bioactive phytochemicals. Int. J. Food Sci. Tech..

[146] Carbonell-Capella J.M., Buniowska M., Barba F.J., Esteve M.J., Frigola A. (2014). Analytical Methods for Determining Bioavailability and Bioaccessibility of Bioactive Compounds from Fruits and Vegetables: A Review. Compr. Rev. Food Sci. Food Saf..

[147] Shin S.A., Joo B.J., Lee J.S., Ryu G., Han M., Kim W.Y., Park H.H., Lee J.H., Lee C.S. (2020). Phytochemicals as Anti-Inflammatory Agents in Animal Models of Prevalent Inflammatory Diseases. Molecules.

[148] D’Archivio M., Filesi C., Vari R., Scazzocchio B., Masella R. (2010). Bioavailability of the polyphenols: Status and controversies. Int. J. Mol. Sci..

[149] Lipinski C.A., Lombardo F., Dominy B.W., Feeney P.J. (2001). Experimental and computational approaches to estimate solubility and permeability in drug discovery and development settings. Adv. Drug. Deliv. Rev..

[150] Yang C.S., Sang S., Lambert J.D., Lee M.J. (2008). Bioavailability issues in studying the health effects of plant polyphenolic compounds. Mol. Nutr. Food Res..

[151] Gao S., Hu M. (2010). Bioavailability challenges associated with development of anti-cancer phenolics. Mini Rev. Med. Chem..

[152] Aqil F., Munagala R., Jeyabalan J., Vadhanam M.V. (2013). Bioavailability of phytochemicals and its enhancement by drug delivery systems. Cancer Lett..

[153] Hertog M.G., Feskens E.J., Hollman P.C., Katan M.B., Kromhout D. (1993). Dietary antioxidant flavonoids and risk of coronary heart disease: The Zutphen Elderly Study. Lancet.

[154] Hertog M.G., Kromhout D., Aravanis C., Blackburn H., Buzina R., Fidanza F., Giampaoli S., Jansen A., Menotti A., Nedeljkovic S. (1995). Flavonoid intake and long-term risk of coronary heart disease and cancer in the seven countries study. Arch. Intern. Med..

[155] Bondonno N.P., Murray K., Cassidy A., Bondonno C.P., Lewis J.R., Croft K.D., Kyro C., Gislason G., Torp-Pedersen C., Scalbert A. (2020). Higher habitual flavonoid intakes are associated with a lower risk of peripheral artery disease hospitalizations. Am. J. Clin. Nutr..

[156] Hammer A., Koppensteiner R., Steiner S., Niessner A., Goliasch G., Gschwandtner M., Hoke M. (2015). Dark chocolate and vascular function in patients with peripheral artery disease: A randomized, controlled cross-over trial. Clin. Hemorheol. Microcirc..

[157] Loffredo L., Perri L., Catasca E., Pignatelli P., Brancorsini M., Nocella C., De Falco E., Bartimoccia S., Frati G., Carnevale R. (2014). Dark chocolate acutely improves walking autonomy in patients with peripheral artery disease. J. Am. Heart Assoc..

[158] McDermott M.M., Criqui M.H., Domanchuk K., Ferrucci L., Guralnik J.M., Kibbe M.R., Kosmac K., Kramer C.M., Leeuwenburgh C., Li L. (2020). Cocoa to Improve Walking Performance in Older People with Peripheral Artery Disease: The COCOA-PAD Pilot Randomized Clinical Trial. Circ. Res..

[159] Clifford T., Howatson G., West D.J., Stevenson E.J. (2015). The potential benefits of red beetroot supplementation in health and disease. Nutrients.

[160] Kenjale A.A., Ham K.L., Stabler T., Robbins J.L., Johnson J.L., Vanbruggen M., Privette G., Yim E., Kraus W.E., Allen J.D. (2011). Dietary nitrate supplementation enhances exercise performance in peripheral arterial disease. J. Appl. Physiol..

[161] Woessner M.N., VanBruggen M.D., Pieper C.F., O’Reilly E.K., Kraus W.E., Allen J.D. (2017). Combined Dietary Nitrate and Exercise Intervention in Peripheral Artery Disease: Protocol Rationale and Design. JMIR Res. Protoc..

[162] Woessner M., VanBruggen M.D., Pieper C.F., Sloane R., Kraus W.E., Gow A.J., Allen J.D. (2018). Beet the Best?. Circ. Res..

[163] Tome-Carneiro J., Gonzalvez M., Larrosa M., Yanez-Gascon M.J., Garcia-Almagro F.J., Ruiz-Ros J.A., Tomas-Barberan F.A., Garcia-Conesa M.T., Espin J.C. (2013). Resveratrol in primary and secondary prevention of cardiovascular disease: A dietary and clinical perspective. Ann. Ny Acad. Sci..

[164] Berman A.Y., Motechin R.A., Wiesenfeld M.Y., Holz M.K. (2017). The therapeutic potential of resveratrol: A review of clinical trials. NPJ Precis Oncol..

[165] McDermott M.M., Leeuwenburgh C., Guralnik J.M., Tian L., Sufit R., Zhao L., Criqui M.H., Kibbe M.R., Stein J.H., Lloyd-Jones D. (2017). Effect of Resveratrol on Walking Performance in Older People with Peripheral Artery Disease: The RESTORE Randomized Clinical Trial. JAMA Cardiol..

[166] Kiesewetter H., Jung F., Jung E.M., Blume J., Mrowietz C., Birk A., Koscielny J., Wenzel E. (1993). Effects of garlic coated tablets in peripheral arterial occlusive disease. Clin. Investig..

[167] Armah C.N., Derdemezis C., Traka M.H., Dainty J.R., Doleman J.F., Saha S., Leung W., Potter J.F., Lovegrove J.A., Mithen R.F. (2015). Diet rich in high glucoraphanin broccoli reduces plasma LDL cholesterol: Evidence from randomised controlled trials. Mol. Nutr. Food Res..

[168] Lopez-Chillon M.T., Carazo-Diaz C., Prieto-Merino D., Zafrilla P., Moreno D.A., Villano D. (2019). Effects of long-term consumption of broccoli sprouts on inflammatory markers in overweight subjects. Clin. Nutr..

[169] Bahadoran Z., Mirmiran P., Hosseinpanah F., Hedayati M., Hosseinpour-Niazi S., Azizi F. (2011). Broccoli sprouts reduce oxidative stress in type 2 diabetes: A randomized double-blind clinical trial. Eur. J. Clin. Nutr..

[170] Bahadoran Z., Mirmiran P., Hosseinpanah F., Rajab A., Asghari G., Azizi F. (2012). Broccoli sprouts powder could improve serum triglyceride and oxidized LDL/LDL-cholesterol ratio in type 2 diabetic patients: A randomized double-blind placebo-controlled clinical trial. Diabetes Res. Clin. Pr..

[171] Bahadoran Z., Tohidi M., Nazeri P., Mehran M., Azizi F., Mirmiran P. (2012). Effect of broccoli sprouts on insulin resistance in type 2 diabetic patients: A randomized double-blind clinical trial. Int. J. Food Sci. Nutr..

[172] Axelsson A.S., Tubbs E., Mecham B., Chacko S., Nenonen H.A., Tang Y., Fahey J.W., Derry J.M.J., Wollheim C.B., Wierup N. (2017). Sulforaphane reduces hepatic glucose production and improves glucose control in patients with type 2 diabetes. Sci. Transl. Med..

[173] Peluso I., Villano Valencia D., Chen C.O., Palmery M. (2020). Antioxidant, Anti-Inflammatory, and Microbial-Modulating Activities of Nutraceuticals and Functional Foods 2019. Oxid. Med. Cell Longev..

[174] Houghton C.A. (2019). Sulforaphane: Its “Coming of Age” as a Clinically Relevant Nutraceutical in the Prevention and Treatment of Chronic Disease. Oxidative Med. Cell. Longev..

[175] Estruch R., Ros E., Salas-Salvado J., Covas M.I., Corella D., Aros F., Gomez-Gracia E., Ruiz-Gutierrez V., Fiol M., Lapetra J. (2018). Primary Prevention of Cardiovascular Disease with a Mediterranean Diet Supplemented with Extra-Virgin Olive Oil or Nuts. N. Engl. J. Med..

[176] Martinez-Gonzalez M.A., Corella D., Salas-Salvado J., Ros E., Covas M.I., Fiol M., Warnberg J., Aros F., Ruiz-Gutierrez V., Lamuela-Raventos R.M. (2012). Cohort profile: Design and methods of the PREDIMED study. Int. J. Epidemiol..

[177] Ruiz-Canela M., Estruch R., Corella D., Salas-Salvado J., Martinez-Gonzalez M.A. (2014). Association of Mediterranean diet with peripheral artery disease: The PREDIMED randomized trial. JAMA.

[178] Tangney C.C., Rasmussen H.E. (2013). Polyphenols, inflammation, and cardiovascular disease. Curr. Atheroscler. Rep..

